# Sodium Hydrosulfide Prevents Myocardial Dysfunction through Modulation of Extracellular Matrix Accumulation and Vascular Density

**DOI:** 10.3390/ijms151223212

**Published:** 2014-12-09

**Authors:** Li-Long Pan, Xian-Li Wang, Xi-Ling Wang, Yi-Zhun Zhu

**Affiliations:** 1 Shanghai Key Laboratory of Bioactive Small Molecules, Department of Pharmacology, School of Pharmacy, Fudan University, Shanghai 201203, China; E-Mails: panlilong@fudan.edu.cn (L.-L.P.); 072103177@fudan.edu.cn (X.-L.W.); xlwang@chempartner.cn (X.-L.W.); 2 Institute of Biomedical Sciences, Fudan University, Shanghai 200032, China

**Keywords:** cardioprotection, hydrogen sulfide, infarction, neovascularization, remodeling

## Abstract

The aim was to examine the role of exogenous hydrogen sulfide (H_2_S) on cardiac remodeling in post-myocardial infarction (MI) rats. MI was induced in rats by ligation of coronary artery. After treatment with sodium hydrosulfide (NaHS, an exogenous H_2_S donor, 56 μM/kg·day) for 42 days, the effects of NaHS on left ventricular morphometric features, echocardiographic parameters, heme oxygenase-1 (HO-1), matrix metalloproteinases-9 (MMP-9), type I and type III collagen, vascular endothelial growth factor (VEGF), CD34, and α-smooth muscle actin (α-SMA) in the border zone of infarct area were analyzed to elucidate the protective mechanisms of exogenous H_2_S on cardiac function and fibrosis. Forty-two days post MI, NaHS-treatment resulted in a decrease in myocardial fibrotic area in association with decreased levels of type I, type III collagen and MMP-9 and improved cardiac function. Meanwhile, NaHS administration significantly increased cystathionine γ-lyase (CSE), HO-1, α-SMA, and VEGF expression. This effect was accompanied by an increase in vascular density in the border zone of infarcted myocardium. Our results provided the strong evidences that exogenous H_2_S prevented cardiac remodeling, at least in part, through inhibition of extracellular matrix accumulation and increase in vascular density.

## 1. Introduction

Peripheral artery disease is an important healthcare problem in the world and is associated with increased risk for coronary artery events, such as myocardial infarction (MI) [1,2]. Adverse cardiac remodeling following MI remains a significant cause of congestive heart failure [3]. Cardiac remodeling is characterized by significant changes in left ventricle size, shape, and function [4]. This myocardial remodeling sets in motion a number of cellular and extracellular matrix events. Particularly, the extracellular matrix turnover in the non-ischemic myocardium is considered to be maladaptive and contributes to the pathophysiology of myocardial remodeling and progression to heart failure [5,6]. In addition, induction and/or activation of matrix metalloproteinases (MMPs) in the surrounding viable myocardium accelerate extracellular matrix turnover and a failure of mature scar formation [7]. Therefore, pharmacological approaches for intervening excessive deposition of extracellular matrix and MMP may be therapeutic strategies for cardiac remodeling.

As a third endogenously produced gaseous signaling molecule, hydrogen sulfide (H_2_S) has emerged as a potentially important mediator in cardiovascular homeostasis and cardioprotection [8,9,10]. H_2_S production has been attributed to three key enzymes in the cysteine biosynthesis pathway, cystathionine β-synthase [11] and cystathionine γ-lyase (CSE), and 3-mercaptopyruvate sulfurtransferase [12,13]. Moreover, CSE is the main H_2_S-generating enzyme that has been identified in cardiovascular system, including heart [14]. H_2_S has seen considerable interest in modulating many physiological and pathophysiological processes [6,15]. Significant reduction of CSE expression or deprivation of endogenous H_2_S generation may lead to the development of various cardiovascular diseases [8,16]. Similar to other gaseous signaling molecules, H_2_S plays several roles in cardiovascular system which include regulation of vessel diameter, protection of endothelium from redox stress, ischemia reperfusion injury and chronic inflammation [17]. The salubrious effects of H_2_S are attributed to its various biological activities, including anti-oxidative [18], anti-inflammatory [19], proangiogenic [15], and vasodilating [20] capacities. Recently, our group reported that the administration of NaHS inhibited apoptosis of myocardial myocytes via protection of mitochondrial function in a model of heart failure [21]. Furthermore, several groups, including our own, have demonstrated that administration of H_2_S played a protective role in the process of fibrosis in the injured myocardium and failing heart [6]. Although the physiological and cardioprotective effects of H_2_S have previously been documented, the effects and molecular mechanisms of H_2_S on remodeling and neovascularization after myocardial ischemia (MI) have not been fully evaluated.

Therefore, the aim of this study was to demonstrate that long-term sodium hydrosulfide (NaHS, a H_2_S donor) [6] therapy impeded MI-induced cardiac dysfunction and extracellular matrix turnover in the non-ischemic myocardium. In addition, we showed that exogenous H_2_S supplementation modulated CSE and HO-1 expression, which contributed to new vessels growth in the border zone of infarct area.

## 2. Results and Discussion

### 2.1. NaHS Inhibited Fibrosis in the Border Zone of Infarcted Myocardium

**Figure 1:**
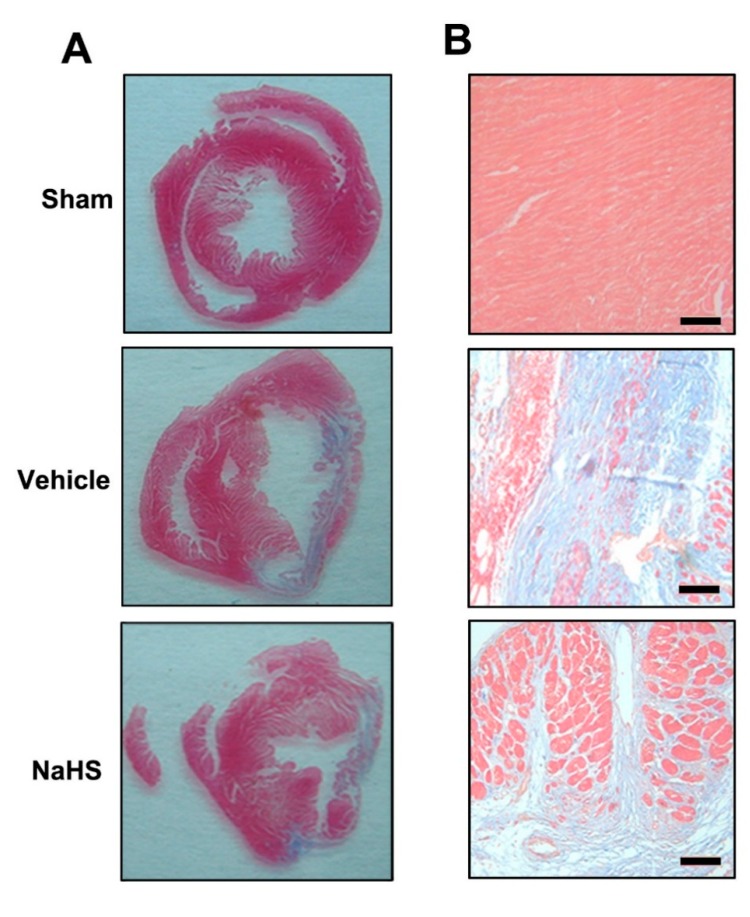

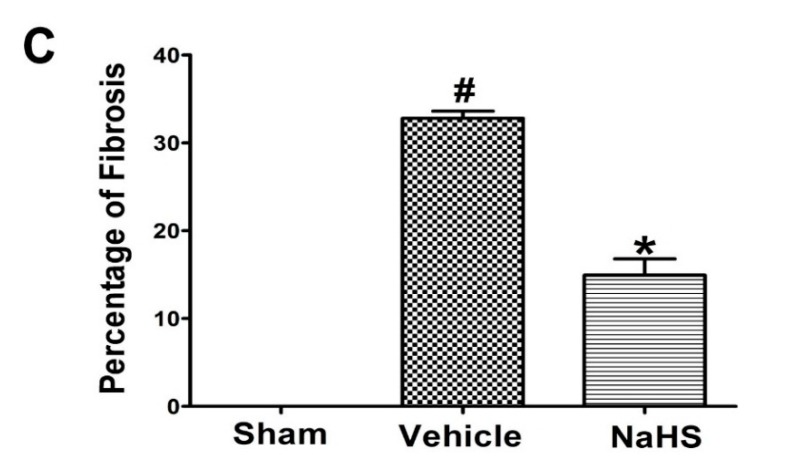
Sodium hydrosulfide (NaHS) inhibited fibrosis in the border zone of infarcted myocardium. (**A**) Representative Masson’s staining of cardiac fibrosis; (**B**) High-magnification microphotographs of Masson-stained sections showed the extent of cardiac fibrosis. Scale bar = 25 μM; and (**C**) Quantitative analysis of fibrosis area (percentage of left ventricular (LV) area) at 42 days post MI. *n* = 6, # *p* < 0.01 *vs*. sham-operated rats; * *p* < 0.01 *vs.* vehicle-treated rats.

Interstitial fibrosis in the border zone of infarcted myocardium is commonly observed in failing hearts and contributes to functional impairment [22]. The degree of fibrosis and chamber remodeling were assessed on 42 days after post MI. As shown in Figure 1A,B, scarce fibrosis of myocardial wall was observed in the sham group. In contrast, significant area of fibrosis occurred in the vehicle group. In agreement with our previous study showing that NaHS administration could reduce the fibrosis size in heart failure rats [21], the extent of fibrosis significantly reduced in the NaHS treatment group (15.0% ± 3.2%) compared with vehicle group (32.8% ± 1.4%) ( 1C, *p* < 0.05). These findings suggested that NaHS might attenuate extracellular matrix accumulation in the border zone of infarcted myocardium.

### 2.2. NaHS Mitigated Type I and III Collagen as well as Matrix Metalloproteinases-9 (MMP-9) Expression

**Figure 2:**
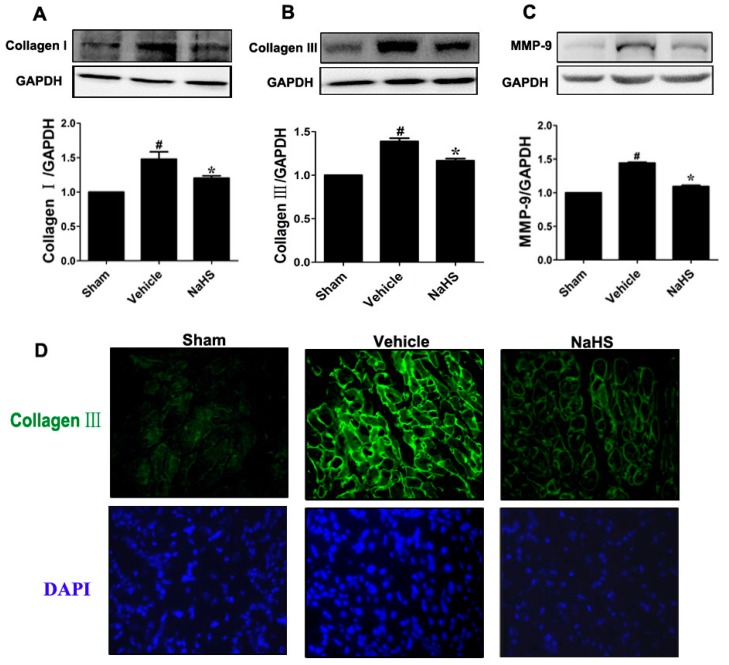
NaHS mitigated type I and III collagen as well as matrix metalloproteinases-9 (MMP-9) expression. Western blot for (**A**) type I collagen; (**B**) type III collagen; and (**C**) MMP-9 expression. Bar graphs showed quantitative analysis of type I collagen, type III collagen, and MMP-9; GAPDH was used as loading control. Representative photomicrographs showing (**D**) type III collagen in the border zone of infarcted myocardium detected by fluorescence microscopy (×400 magnification). # *p* < 0.05 *vs*. sham-operated group; * *p* < 0.05 *vs*. vehicle-treated group; *n* = 6.

Excessive extracellular matrix accumulation in the non-ischemic myocardium leads to myocardial fibrosis and ventricular dysfunction [23]. In agreement with previous findings that chronic MI induced myocardial fibrosis in rats 42 days post MI, the levels of collagen types I and III protein in the border zone of infarcted tissues were increased by 35% and 47% compared to sham group, respectively. NaHS treatment was associated with significant decreased collagen type I and type III expression in the border zone of infarcted tissues (14%) at 42 days post MI (Figure 2A,B, *p* < 0.05). Meanwhile, the effects of NaHS administration on collagen types III expression were also confirmed by immunofluorescence staining through specific antibodies (Figure 2C). Consistent with the protein expression by Western bot analysis, the cross-sectional views showed relative low level of collagen type III (bright, wavy appearance) presents in the border zone of infarcted tissues in those sections of sham rats. However, vehicle-treated MI rats exhibited marked increased collagen type III protein accumulation, which was significantly reduced by rats treated with NaHS. Furthermore, we also measured MMP-9 level, which was associated with an improvement of myocardial remodeling. As shown in Figure 2D, MMP-9 expression in vehicle-treated animals marked increased compared with sham-operated rats, whereas NaHS administration significantly attenuated this increase (*p* < 0.05).

### 2.3. NaHS Modulated CSE and Heme Oxygenase-1 Expression in the Border Zone of Infarcted Myocardium

**Figure 3:**
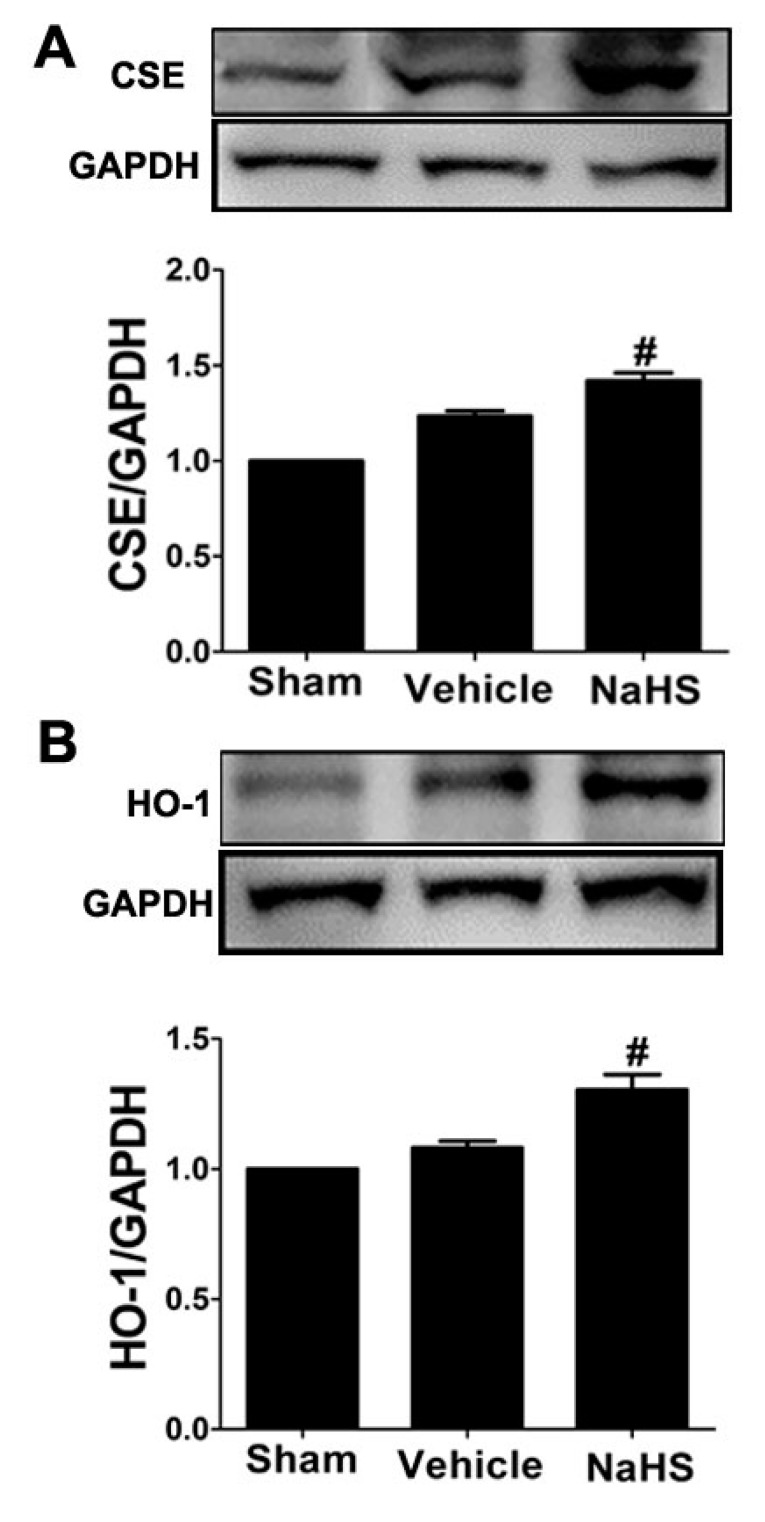
NaHS modulated cystathionine γ-lyase (CSE) and heme oxygenase-1 (HO-1) expression in the border zone of infarcted myocardium. Western blot for (**A**) CSE and (**B**) HO-1 expression. Bar graphs showed quantitative analysis of CSE and HO-1; GAPDH was used as loading control. # *p* < 0.05 *vs*. sham-operated rats; *n* = 6.

Our previous study has shown that NaHS inhibited fibrotic response *in vivo* and *in vitro* via upregulation of heme oxygenase-1 (HO-1) [6]. To investigate the underlying mechanism of H_2_S on chronic-induced cardiac fibrosis, we examined the effects of NaHS on HO-1 expression in the border zone of infarcted tissues. Consistent with our previous report [6], therapy with NaHS significantly induced HO-1 expression compared with sham-operated rats (*p* < 0.05), but no significant change in HO-1 expression between sham-operated or vehicle-treated animals was observed (*p* < 0.05) (Figure 3A). We previously reported that a reduction in H_2_S plasma and tissue levels was observed in ischemic heart disease [9,21], therefore, the expression of H_2_S-generating enzyme CSE was also analyzed by western blot analysis. As shown in Figure 3B, a slight increase CSE expression in the border zone of infarcted tissues was observed in vehicle-treated animals compared to the sham-operated rats (*p* < 0.05). Interestingly, NaHS therapy markedly increased CSE protein expression in the border zone of infarcted tissues compared with sham-operated rats (*p* < 0.05).

### 2.4. NaHS Promoted the Growth of New Vessels in the Border Zone of Infarcted Myocardium

**Figure 4:**
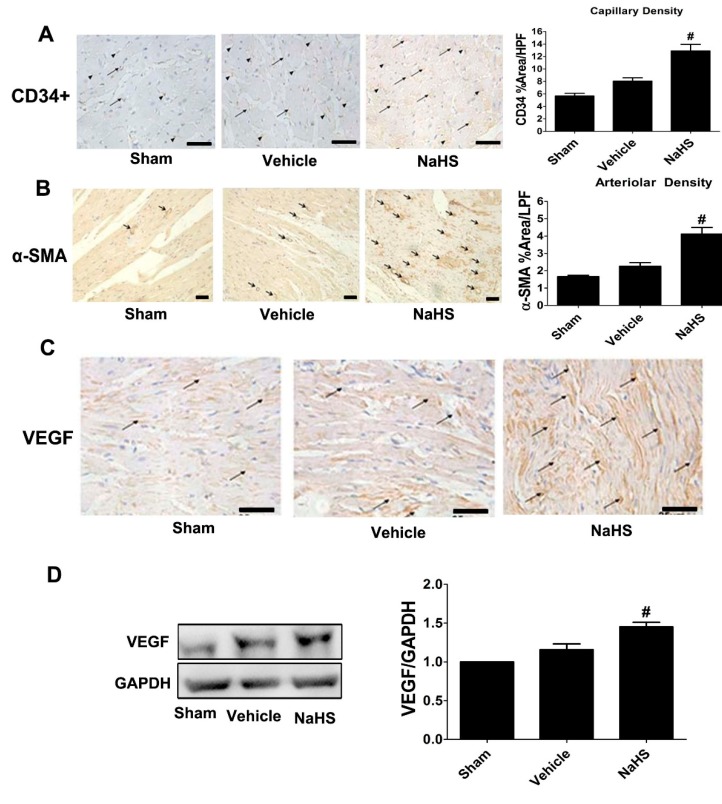
NaHS promoted the growth of new vessels in the border zone of infarcted myocardium. Representative photomicrographs showing capillary density (CD34 staining, black arrow and arrowheads indicated capillaries) (**A**); arteriolar density (α-SMA staining, black arrow indicated arteries) (**B**); and vascular endothelial growth factor (VEGF) protein (black arrow indicated VEGF expression) (**C**) in the border zone of infarcted myocardium detected by immunohistochemical staining. Scale bar = 25 μM; HPF, ×20 high-powered field; LPF, ×10 low-powered field; and (**D**) Western blot for VEGF expression. Bar graphs showed quantitative analysis of VEGF; GAPDH was used as loading control. # *p* < 0.05 *vs.* sham-operated rats; *n* = 6.

Immunohistochemical staining of CD34 was carried out to assess the capillary density in theborder zone of the infarct area. As shown in Figure 4A capillary density (CD34 cells) in the border zone of infarct area slightly increased compared with sham-operated animals. Interestingly, NaHS treatment significantly increased capillary density compared to vehicle group (Figure 4A). Meanwhile, NaHS administration, but not vehicle treatment, also markedly enhanced arteriolar density in the border zone of infarct area compared with sham-operated rats by immunohistochemical staining using specific artery marker antibody α-smooth muscle actin (α-SMA) (Figure 4B). Because vascular endothelial growth factor (VEGF) is a pivotal angiogenic factor, we next examined VEGF expression in the border zone of infarct area with or without the administration of NaHS using immunohistochemical staining and Western blot. NaHS, but not vehicle treatment, significantly augmented VEGF level in the border zone of infarct area compared with sham-operated rats as measured by Western blot (Figure 4C, *p* < 0.05). In addition, the similar VEGF expression in the border zone of infarct area among three groups was also identified by immunohistochemical staining (Figure 4D).

### 2.5. NaHS Prevented Cardiac Dysfunction

**Figure 5:**
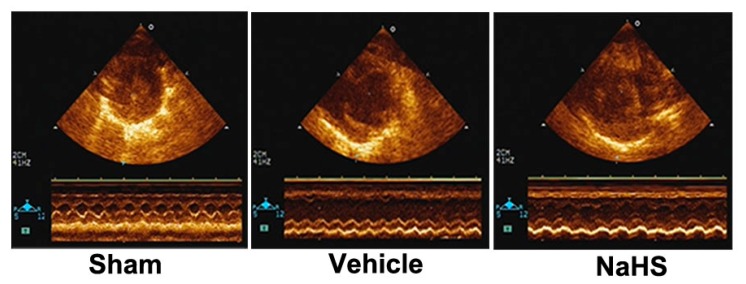
NaHS prevented cardiac dysfunction. Echocardiography assessment of cardiac function. Representative images of echocardiography. *n* = 7.

The echocardiographic data was shown in Figure 5 and Table 1, 42 days post MI coursed chamber dilatation as detected by increased left ventricular internal dimension diastole (LVIDd) and left ventricular internal dimension systole (LVIDs) (*p* < 0.05) and cardiac dysfunction as assessed by decreased percentage of fractional shortening (FS) and ejection fraction (EF) (*p* < 0.05). However, NaHS therapy markedly prevented chamber dilatation and attenuated cardiac dysfunction as evidenced by increased percentage of FS and EF (*p* < 0.05). Heart rates were not significantly different among three groups.

**Table 1:** Echocardiographic Parameters.

Parameters	Sham (*n* = 7)	Vehicle (*n* = 8)	NaHS (*n* = 8)
LVIDd (cm)	0.57 ± 0.02	0.73 ± 0.03 #	0.71 ± 0.03
LVIDs (cm)	0.3 ± 0.02	0.51 ± 0.06 #	0.41 ± 0.05 *
R-R (s)	0.15 ± 0.01	0.15 ± 0.01	0.16 ± 0.01
LVEDV (mL)	0.42 ± 0.04	0.88 ± 0.05 #	0.81 ± 0.14
LVESV (mL)	0.07 ± 0.01	0.35 ± 0.06 #	0.19 ± 0.05 *
EF (%)	84.0 ± 0.30	63.0 ± 0.20 #	78.0 ± 0.30 *
FS (%)	47.37 ± 3.44	28.55 ± 1.17 #	39.45 ± 3.11 *
HR (bpm)	393 ± 16	415 ± 16	396 ± 13

LVIDd, left ventricular internal dimension diastole; LVIDs, left ventricular internal dimension systole; R-R, R-R intervals; LVEDV, left ventricular end-diastolic volume; LVESV, left ventricular end-systolic volume; EF, ejection fraction; FS, fractional shortening; HR, heart rate; # *p* < 0.05 *vs.* sham-operated animals; * *p* < 0.05 *vs*. vehicle-treated rats.

### 2.6. Discussion

The results presented in this study demonstrated that supplementation with exogenous H_2_S attenuated myocardial remodeling and prevented cardiac dysfunction post MI. These structural and functional changes were associated with reduced extracellular matrix accumulation (types I and III collagen, MMP-9 expression) and induced new vessels growth. In addition, modulation of CSE and HO-1 levels by exogenous H_2_S supplementation might contribute to anti-fibrotic and pro-angiogenic effects in the border zone of infarct area.

Several groups, including our own, have demonstrated that both exogenously H_2_S and endogenously derived H_2_S exhibit potent cardioprotective effects in models of acute MI and ischemia-induced heart failure [6,21,24,25]. However, the role and mechanism of H_2_S in ischemia-induced myocardial remodeling remains elusive. In our previous studies, endogenous H_2_S level in plasma significantly decreased after acute MI [26]. Exogenous H_2_S supplementation exerted cardioprotective effects through modulation of intracellular redox balance and reduction of cardiomyocytes loss during acute MI [27]. Recently, various studies have demonstrated that lower circulating H_2_S levels suffering from cardiac remodeling correlated negatively with the severity of the disease in models of heart failure or in patients [24,28]. However, in concert with previous studies [6,21], a slight increase of CSE expression was observed in the chronic ischemia myocardium in the present study. Meanwhile, we found that exogenous H_2_S therapy attenuated cardiac dysfunction and remodeling post MI. These findings are consistent with previous studies, which demonstrated H_2_S therapy attenuated left ventricular remodeling and dysfunction [21,24]. The upregulation of CSE in chronic ischemia myocardium may thus be a compensatory attempt to counterregulate the decreased level of endogenous H_2_S in chronic ischemia myocardium [10]. Herein, we found that exogenous H_2_S therapy modulated CSE expression in the border zone of infarct area and finally prevented cardiac remodeling and modulated CSE expression, which indicated that modulation of CSE expression contributed to attenuate cardiac remodeling post MI by inducing angiogenesis and directly inhibiting cardiac fibroblasts activation [6,24]. The mechanisms involved in the post MI myocardial remodeling may include complex pathways other than the CSE/H_2_S-mediated process. The above effects were suggested to be partly attributable to H_2_S-mediated compromised systemic changes, which may affect the disease process or outcome.

Excessive deposition of extracellular matrix, predominantly collagen, in peri-infarcted area represents myocardial stiffness leading to relaxation abnormalities and progressive dysfunction culminating in heart failure [29,30]. Therefore, reduction of excessive interstitial fibrosis in border infarcted area exerts a beneficial role in cardiac remodeling post MI [6]. Meanwhile, MMP-9 is involved in extracellular matrix degradation and cardiac remodeling and MMP-9 inhibition improves outcomes [3,31]. Activation post-infarction inflammation may promote cardiac remodeling through several distinct mechanisms, such as enhancing MMP activities, and increasing matrix degradation [32]. Our previous findings have demonstrated that H_2_S not only exerted anti-inflammatory activity but also inhibited pro-fibrotic response under acute MI in rats [6,21]. To further confirm whether exogenous H_2_S inhibited myocardial fibrosis in the border zone of infarct area, we examined the effects of exogenous H_2_S on extracellular matrix (types I and III collagen) deposition and MMP-9 expression, which play critical roles in the process of myocardial remodeling post MI. Intriguingly, our results demonstrated that pharmacologic supplementation of exogenous H_2_S substantially inhibited types I and III collagen as well as MMP-9 expression in peri-infarct area after MI. It was associated with decreased myocardial fibrosis and prevented cardiac dysfunction. Therefore, inhibition of extracellular matrix accumulation and MMP-9 expression by exogenous H_2_S might contribute to its anti-fibrotic effect after MI.

H_2_S is also a pro-angiogenic agent and promotes new vessel formation in the setting of ischemic diseases [33,34,35]. VEGF, a potent angiogenic cytokine, plays a central role in coronary vascular network growth under ischemic conditions [36]. In the present study, exogenous H_2_S therapy increased VEGF expression post MI. This was associated with increased vascular density in the exogenous H_2_S-treated hearts, as evidenced by the increase in the expression of CD34 and α-SMA. Various studies have reported that HO-1 induction is an important cardioprotective adaptation that opposes pathological myocardial remodeling through production of its metabolites such as carbon monoxide, bilirubin, and free iron [6,37,38]. HO-1 has also been reported to play a role in mediating VEGF-induced angiogenesis [39,40]. Although HO-1 and H_2_S signaling have traditionally been considered to operate via distinct pathways, there is evidence of cross-talk between the two pathways. For instance, The HO-1 positively modulates VEGF synthesis and promotes angiogenesis via activation of several intracellular signal pathways [41]. While, the increase in VEGF expression is amplified via a positive feedback loop, where VEGF-induced S-nitrosylation of Keap1 promotes Nrf2-dependent HO-1 induction [26,42]. In agreement with our previous reports [6,19], we found that exogenous H_2_S therapy dramatically induced HO-1 expression in the border zone of the infarct area. We also found that the upregulation of HO-1 by exogenous H_2_S partly contributed to ameliorating detrimental remodeling, as evidenced by reducing extracellular matrix accumulation and impeding cardiac dysfunction. Obviously, both biological molecules do not always work independently, but rather can cross-modulate each other’s biological activity in the vasculature. Although, a slight increase in VEGF and HO-1 expression and new vascular formation were observed in vehicle-treated hearts, which might be due to negative feedback caused by the reparative mechanisms of the body. These evidences suggested that exogenous H_2_S stimulated the angiogenic processes post MI via a positive feedback loop between HO-1 and VEGF axes. This effect was accompanied by an increase in vascular density and reduced myocardial fibrosis in the border zone of infarct area. Collectively, the induction of angiogenesis via VEGF-HO-1 signaling afforded by H_2_S contributed to prevent the adverse remodeling and improve cardiac function post MI.

## 3. Methods

### 3.1. Animal Care

All animals used in this study received humane care and in compliance with the principles of laboratory animal care formulated by Fudan University for Medical Research and Guide for the Care and Use of Laboratory Animals.

### 3.2. Chemicals and Antibodies

NaHS was purchased from Sigma–Aldrich (St. Louis, MO, USA). Antibodies against GAPDH, VEGF, HO-1, and CSE were from Santa Cruz Biotechnology (Santa Cruz, CA, USA); antibodies against collagen type I and type III, MMP-9 were purchased from Calbiochem (Dermstadt, Germany); antibodies against CD34 and α-SMA were from Abcam (Shanghai, China).

### 3.3. Generation of Chronic MI and Drug Treatment

Male Sprague-Dawley rats weighing between 200 and 250 g were anesthetized with sodium pentobarbital (50 mg/kg i.p. (intraperitoneal injection)). MI was induced in rats by ligation of left anterior descending artery (LAD) as described previously [6,22]. Rats were either subjected to left coronary artery ligation (*n* = 30) or sham surgery (*n* = 10). During ligation of the LAD, rats were randomized to one of two groups; Vehicle group: this group consisted of rats with myocardial ischemia that received saline (*n* = 15); NaHS group: MI rats infused with NaHS (56 μM/kg·day, *n* = 15). All treatments were intraperitoneally injected after LAD and were continued thereafter (once per day for seven days per week). The experimental period was 6 weeks. NaHS was employed as an exogenous H_2_S donor and the dose used in the present study was selected on the basis of our previous publications [6,21].

### 3.4. Echocardiography

Echocardiographic studies were performed at 42 days after operation. The rat was lightly anesthetized with sodium pentobarbital (50 mg/kg, i.p.), and transthoracic echocardiography was performed using an echocardiographic system (SONOS 7500; Philips Medical System, Best, The Netherlands) equipped with a 12.0-MHz phased-array transducer. Two-dimensional short-axis views of the left ventricle and M-mode tracings were recorded to measure LVIDd, LVIDs, left ventricular end-diastolic volume (LVEDV), left ventricular end-systolic volume (LVESV), EF, and FS.

### 3.5. Sample Preparation for Histological and Morphometric Analysis

After cardiac function evaluation, hearts were collected and embedded in OCT compound (Tissue Tek; Miles Inc., Elkhart, IN, USA), frozen in liquid nitrogen-cooled and stored at −80 °C. The samples were sectioned to a thickness of 5 µm. Percentage of fibrosis was determined with Masson’s trichrome stained tissue sections using Image J software (National Institutes of Health, Bethesda, MD, USA).

### 3.6. Immunohistochemistry and Immunofluorescence Analysis

The samples from 42-day treatment (*n* = 6 in each group) were fixed with 4% buffered formalin, and embedded in paraffin. Fibrosis area and total LV area were expressed as the percentage of the area of fibrosis. To assess neovascularization, cryosections from 42-day samples were incubated with antibodies against VEGF, CD34, and α-SMA, respectively. The slides were washed and incubated with biotinylated, affinity-purified goat anti-mouse IgG as the secondary antibody. After avidin-biotin amplification, the slides were incubated with 3,3'-diaminobenzidine and counterstained with hematoxylin. Sections were examined with a microscope (Nikon ECLIPSE TE200, Tokyo, Japan). Three representative microscopic images of each sections were digitally captured and analyzed for capillary density (CD34-positive structures) and arteriolar density (α-SMA-positive structures) and analyzed using Image J software.

For immunofluorescence staining, the sections were blocked with 5% BSA for 1 h and incubated with the primary antibody against collagen type III. Subsequently, sections were incubated with fluorescein isothiocyanate conjugated anti-rabbit antibody (Invitrogen, Carlsbad, CA, USA), and counterstained for nuclei with DAPI. Immunofluorescence was visualized using a fluorescent microscope (Carl Zeiss Inc., Jena, Germany). The results were based on three independent analyses.

### 3.7. Western Blot Analysis

The border zone of infarcted tissues (defined as 1 mm of myocardium surrounding the infarct zone) was isolated as described elsewhere [21,22]. Protein concentration was determined with a BCA protein assay kit (Biocolor Biotechnology, Shanghai, China). Western blot analysis was carried out as our previously described elsewhere [6]. Equal amounts (30 μg) of protein were separated on SDS-polyacrylamide gels, transferred to a nitrocellulose membrane (GE Healthcare, Buckingham, UK), and incubated with the appropriate primary antibodies in 5% fat-free milk in Tris-buffered saline with 0.1% Tween, followed by HRP-conjugated anti-mouse or anti-rabbit IgG (1:5000; ICL Lab, Newberg, OR, USA). The immunoreactive bands were visualized by enhanced chemoluminescence with a camera-based imaging system (Alpha Innotech, Santa Clara, CA, USA). The density of the signals was quantified with the AlphaEase software. The primary antibodies were used as follows: CSE (1:500), HO-1(1:1000), VEGF (1:500), Collagen I (1:500), Collagen III (1:500), MMP-9 (1:1000), and GAPDH (1:2000).

### 3.8. Statistical Analysis

Data were expressed as mean ± standard error of the mean (mean ± SEM). All data analysis was performed with the use of GraphPad Prism 5 software (GraphPad-Prism Software Inc., San Diego, CA, USA). Differences between mean values of multiple groups were analyzed by one-way analysis of variance with Tukey’s test for *post hoc* comparisons. Statistical significance was considered at *p* < 0.05.

## 4. Conclusions

In summary, this study highlights the role of exogenous H_2_S supplementation in MI-induced myocardial remodeling and cardiac dysfunction, and has shown that exogenous H_2_S therapy suppresses extracellular matrix accumulation and induces new vessels growth in the border zone of infarct area, at least in part, through modulation of HO-1 and CSE expression. The cardioprotective effect of exogenous H_2_S suggests that H_2_S supplementation may be a promising candidate for ventricular remodeling post MI.

## Abbreviation

CSE: cystathionine γ-lyase
MI: myocardial infarction
MMPs: matrix metalloproteinases
HO-1: heme oxygenase-1
VEGF: vascular endothelial growth factor
NaHS: Sodium hydrosulfide
H_2_S: Hydrogen sulfide
LVIDd: left ventricular internal dimension diastole
LVIDs: left ventricular internal dimension systole
LVEDV: left ventricular end-diastolic volume
LVESV: left ventricular end-systolic volume
EF: ejection fraction
FS: fractional shortening
